# Postoperative Pneumonia After Minimally Invasive Esophagectomy in Esophageal Squamous Cell Carcinoma: A Weighted Real-World Comparison of Neoadjuvant Treatment Strategies

**DOI:** 10.3390/cancers18162571

**Published:** 2026-08-10

**Authors:** Funa Yang, Tao Zhang, Wei Wei, Xiaocan Jia, Lanwei Guo, Fengjuan Liu, Peinan Chen, Haibo Sun, Hongying Shi, Wei Cui

**Affiliations:** 1 Department of Hospital Infection Control, The Affiliated Cancer Hospital of Zhengzhou University & Henan Cancer Hospital, Zhengzhou 450008, China; zlyyyangfuna3358@zzu.edu.cn (F.Y.);; 2 Department of Epidemiology and Biostatistics, College of Public Health, Zhengzhou University, Zhengzhou 450001, China; 3 Department of Clinical Research Management, The Affiliated Cancer Hospital of Zhengzhou University & Henan Cancer Hospital, Zhengzhou 450008, China; 4 Prevention Medicine Department, Henan Chest Hospital, Zhengzhou 450003, China; 5 Department of Thoracic Surgery, The Affiliated Cancer Hospital of Zhengzhou University & Henan Cancer Hospital, Zhengzhou 450008, China; chenpeinan0127@zzu.edu.cn (P.C.); zlyysunhaibo2122@zzu.edu.cn (H.S.); 6 Office of the Director, The Affiliated Cancer Hospital of Zhengzhou University & Henan Cancer Hospital, Zhengzhou 450008, China; zlyyshihongying4766@zzu.edu.cn; 7 Department of Clinical Laboratory, The Affiliated Cancer Hospital of Zhengzhou University & Henan Cancer Hospital, Zhengzhou 450008, China; 8 National Cancer Center/National Clinical Research Center for Cancer/Cancer Hospital, Chinese Academy of Medical Sciences and Peking Union Medical College, Beijing 100021, China

**Keywords:** esophageal squamous cell carcinoma, neoadjuvant therapy, postoperative pneumonia, minimally invasive esophagectomy, overlap weighting

## Abstract

**Background**: Postoperative pneumonia (POP) is a critical complication after minimally invasive esophagectomy (MIE) for esophageal squamous cell carcinoma (ESCC). However, the comparative odds of POP among different neoadjuvant treatment strategies remain controversial. This study aimed to compare the associations between different treatment strategies and POP in this population. **Methods**: A retrospective cohort of ESCC patients undergoing MIE between March 2021 and July 2025 was analyzed. Patients were divided into four groups: the surgery alone (S-alone) group (*n* = 282), the neoadjuvant chemotherapy (NCT) group (*n* = 127), the neoadjuvant chemoradiotherapy (NCRT) group (*n* = 60), and the neoadjuvant chemoimmunotherapy (NCIT) group (*n* = 71). To address baseline confounding, generalized overlap weighting (GOW) was used for the four-group comparison, and overlap weighting (OW) was used for the pairwise comparisons of NCRT versus NCT and NCIT versus NCT. Logistic regression models were used to evaluate the associations between treatment strategies and the odds of POP. Sensitivity analyses of the pairwise comparisons were performed using stabilized inverse probability of treatment weighting (S-IPTW). **Results**: In the four-group comparison, neither NCT nor NCRT was associated with significantly higher odds of POP than S-alone, whereas NCIT was associated with higher odds (weighted odds ratio [*OR*], 1.95; 95% confidence interval [*CI*], 1.02–3.70). In pairwise analyses, the weighted ORs were 0.45 (95% *CI*, 0.20–1.01) for NCRT versus NCT and 2.32 (95% *CI*, 1.14–4.71) for NCIT versus NCT. Sensitivity analyses were directionally consistent. **Conclusions**: In this single-center observational cohort, NCRT was not associated with higher odds of POP than S-alone or NCT, whereas NCIT was associated with higher odds of POP. These findings support careful perioperative respiratory management in patients receiving neoadjuvant immunotherapy.

## Simple Summary

This retrospective study used overlap weighting to compare the odds of postoperative pneumonia (POP) after minimally invasive esophagectomy across treatment strategies for esophageal squamous cell carcinoma. Neoadjuvant chemoimmunotherapy was associated with higher odds of POP than surgery alone or neoadjuvant chemotherapy, whereas neoadjuvant chemoradiotherapy was not. These findings support careful perioperative respiratory management in patients receiving neoadjuvant chemoimmunotherapy.

## 1. Introduction

Globally, esophageal cancer (EC) continues to present a major public health challenge, standing as the eleventh most prevalent malignancy and the seventh most frequent cause of oncology-related deaths [1]. Esophageal squamous cell carcinoma (ESCC) is the predominant histological subtype in China [2], representing more than 50% of global cases and related deaths [3,4]. While minimally invasive esophagectomy (MIE) serves as the surgical cornerstone, surgery alone (S-alone) is insufficient for locally advanced ESCC [5]. Consequently, neoadjuvant therapy with MIE has become the standard of care. Over time, these neoadjuvant strategies have evolved from neoadjuvant chemotherapy (NCT) and neoadjuvant chemoradiotherapy (NCRT) to neoadjuvant chemoimmunotherapy (NCIT), which has demonstrated promising pathological responses in resectable ESCC [6,7].

Postoperative pneumonia (POP) is recognized as a common and severe complication following esophagectomy, exerting a profound impact on both short-term recovery and long-term survival [8,9]. Although MIE may reduce surgical trauma compared with open esophagectomy, postoperative pulmonary complications remain clinically important [10,11]. Previous studies have shown that POP is associated with longer hospital stays, higher medical costs, increased perioperative mortality, and poorer long-term oncological outcomes, including overall survival (OS) [12,13,14].

Different neoadjuvant strategies may be associated with differences in the odds of POP through their distinct effects on pulmonary tissues, including radiation-induced damage from NCRT [15] and immune-related lung vulnerability from NCIT [16,17]. However, outcomes from randomized controlled trials (RCTs), real-world cohorts, and meta-analyses assessing the association between neoadjuvant strategies and POP remain inconsistent. A meta-analysis reported that pulmonary complications were significantly increased by NCRT compared to NCT [18], whereas Tang et al. reported comparable perioperative safety between the two regimens [19]. Whether NCIT is associated with higher odds of POP than NCT remains uncertain, as recent studies have reported inconsistent findings regarding perioperative safety [20,21].

These discrepancies might be explained by population heterogeneity, residual confounding, differences in outcome definitions, and limitations of conventional causal inference approaches. Propensity score matching (PSM) may reduce the effective sample size, whereas inverse probability of treatment weighting (IPTW) can be sensitive to extreme weights [22]. Therefore, overlap weighting was adopted to improve covariate balance while reducing the influence of extreme propensity scores [23].

This study aimed to evaluate the associations between treatment strategies and POP in patients with ESCC using generalized overlap weighting (GOW) and overlap weighting (OW), with the goal of informing preoperative decision-making, patient selection, and perioperative respiratory management.

## 2. Materials and Methods

### 2.1. Study Design and Participants

A total of 589 patients who underwent minimally invasive esophagectomy (MIE) for esophageal squamous cell carcinoma (ESCC) at a tertiary hospital in Henan Province between March 2021 and July 2025 were identified. The inclusion and exclusion criteria for this study are presented in Figure S1. Patients who underwent two-lung ventilation were excluded because their intraoperative respiratory management differed substantially from the standard one-lung ventilation protocol, which may independently affect postoperative pulmonary complications [24]. After exclusions, 540 patients were included in the final analysis. Among them, 282 received surgery alone (S-alone), 127 received neoadjuvant chemotherapy (NCT), 60 received neoadjuvant chemoradiotherapy (NCRT), and 71 received neoadjuvant chemoimmunotherapy (NCIT). This study was approved by the Ethics Committee of Zhengzhou University (Approval No.: ZZUIRB2022-99) for all data collection and analysis.

All clinical data were obtained from the hospital’s electronic medical record system. The collected variables included the calendar period of surgery (2021–2022 vs. 2023–2025), demographic characteristics (age, sex, and body mass index [BMI]), smoking, comorbidities (diabetes, hypertension, and pulmonary disease), and nutritional status assessed using the Nutritional Risk Screening 2002 (NRS-2002). Tumor characteristics (clinical tumor stage and tumor location), preoperative treatments (chemotherapy, radiotherapy, and immunotherapy), surgery-related variables (surgical anastomosis, operative duration, and intraoperative blood loss), and the primary outcome of postoperative pneumonia were also collected. The NRS-2002 evaluates nutritional impairment, disease severity, and age, with higher scores indicating greater nutritional risk.

### 2.2. Neoadjuvant Treatment

Patients were treated with neoadjuvant therapy following standard institutional protocols in accordance with Chinese clinical guidelines for esophageal cancer. Neoadjuvant chemotherapy included platinum-based doublet chemotherapy, primarily consisting of taxanes (paclitaxel or docetaxel) combined with a platinum agent (cisplatin, carboplatin, or nedaplatin). Radiotherapy was conducted concurrently with chemotherapy, typically delivering a total dose of 41.4 Gy in 23 fractions. Neoadjuvant chemoimmunotherapy consisted of chemotherapy combined with programmed cell death protein 1 (PD-1) inhibitors. Detailed drug combinations, PD-1 inhibitors and treatment cycles are reported in Supplementary Table S1. After the completion of neoadjuvant therapy, all eligible patients underwent MIE.

### 2.3. Perioperative Management

A formal enhanced recovery after surgery (ERAS) program was not implemented during the study period; however, all patients were managed according to a unified departmental perioperative clinical pathway for radical esophagectomy. Preoperative pulmonary function data were available for the majority of patients. Before study initiation, each operating surgeon had independently performed more than 200 minimally invasive esophagectomies, reflecting substantial experience with the procedure [25].

### 2.4. Definition of Postoperative Pneumonia (POP)

Postoperative pneumonia (POP) was defined as pneumonia occurring within 30 days after surgery according to the Chinese Expert Consensus on the Prevention and Control of Postoperative Pneumonia [26]. Diagnosis required all three of the following criteria: (1) an imaging criterion: at least two chest radiographs, or one chest radiograph in patients without underlying cardiac or pulmonary disease, showing new or progressive and persistent pulmonary infiltrates, consolidation, or cavitation; (2) a systemic criterion: at least one of fever (body temperature > 38 °C) without another identified cause, a peripheral white blood cell count > 12 × 10^9^/L or <4 × 10^9^/L, or altered mental status without an identifiable cause in patients aged ≥ 70 years; and (3) a respiratory criterion: at least two of new-onset purulent sputum or a change in sputum characteristics, increased respiratory secretions, an increased frequency of sputum suctioning, new or worsening cough, dyspnea, or tachypnea, pulmonary rales or bronchial breath sounds, worsening gas exchange, increased oxygen requirements, or the need for mechanical ventilation. POP was diagnosed during routine postoperative care by the treating clinical team and documented in the electronic medical record. For this retrospective cohort, POP-related information was extracted from the medical records and verified against the predefined criteria. The same diagnostic window and criteria were applied to all treatment groups.

### 2.5. Statistical Analysis

Continuous variables were expressed as medians alongside interquartile ranges (IQRs) and compared via the Wilcoxon rank-sum or Kruskal–Wallis tests due to their nonnormal distribution. Categorical variables were displayed as absolute counts and percentages, with statistical differences evaluated through Pearson’s chi-squared test or Fisher’s exact test.

The association between treatment strategy and POP was evaluated in three steps:

(1) Overall comparison across the four treatment groups.

The S-alone group served as the reference. To address baseline imbalances across multiple groups, a generalized propensity score was first estimated using a multinomial logistic regression model incorporating sex, age, year of surgery, BMI, smoking, lung disease, diabetes, hypertension, NRS-2002, clinical tumor stage and tumor location. Covariates were selected based on clinical relevance and their potential associations with both neoadjuvant treatment selection and postoperative pneumonia, rather than according to univariable statistical significance. All variables included in the propensity score model were measured before treatment assignment or were available at the time of treatment decision-making. Year of surgery was included to account for potential temporal changes in treatment selection and perioperative practice. For the four-group comparison, generalized overlap weights were calculated as $w_{i}=\{1/e_{T_{i}}/\sum_{k=1}^{K}\{1/e_{k}(X_{i})\}$, where $e_{k}(X_{i})$ denotes the generalized propensity score for treatment group *k* [27]. Subsequently, three logistic regression models were constructed. Model 1 only included treatment group in the unweighted cohort. Model 2 further adjusted for sex, age, year of surgery, BMI, smoking, lung disease, diabetes, hypertension, NRS-2002, clinical tumor stage, tumor location, surgical anastomosis, operative duration and intraoperative blood loss and Model 3 was a generalized-overlap-weighted multivariable logistic regression model adjusted for all covariates included in Model 2.

(2) Comparison between NCRT and NCT.

The NCT group was set as the reference group. We adopted binary logistic regression to calculate propensity scores for weight assignment, followed by OW. Weights were determined according to the probability of receiving the alternative treatment. In this pairwise comparison, overlap weights were assigned as $1-e(X_{i})$ for patients in the treated group and $e(X_{i})$ for patients in the reference group [28]. For pairwise comparison, three corresponding logistic regression models were fitted: a univariable model, a multivariable model, and an OW-weighted model.

(3) Comparison between NCIT and NCT.

The NCT group also served as the reference for the NCIT versus NCT comparison, and the same analytical procedures were applied.

Overlap weighting was selected because it retains all eligible patients while emphasizing those with comparable probabilities of receiving the alternative treatment strategies. In contrast, propensity score matching may exclude unmatched patients and reduce statistical precision, whereas conventional inverse probability of treatment weighting may generate large or unstable weights in patients with extreme propensity scores. Overlap weights are bounded and therefore generally less sensitive to limited propensity score overlap and extreme weights [23].

Covariate balance was assessed before and after weighting using standardized mean differences (SMDs). For the four-group comparison, the maximum pairwise SMD across treatment groups was reported, whereas absolute SMDs were used for pairwise comparisons. An SMD < 0.10 indicated good covariate balance, whereas an SMD < 0.20 was considered acceptable balance [29]. The weighted analyses relied on the assumptions of conditional exchangeability, positivity, consistency, and correct specification of the propensity score models.

### 2.6. Sensitivity Analysis

As a sensitivity analysis for the two pairwise comparisons, stabilized inverse probability of treatment weighting (S-IPTW) was applied using the same set of covariates as those included in the overlap weighting models to address measured confounding. The results obtained from the S-IPTW-weighted models were compared with those from the primary OW analyses to assess the robustness of the findings.

All analyses were conducted using R software (version 4.5.1; R Foundation for Statistical Computing, Vienna, Austria). The R packages “WeightIt”, “cobalt”, and “survey” were used. Statistical significance was defined as a *p*-value of less than 0.05.

### 2.7. Subgroup Analysis

Subgroup analyses using overlap weighting were performed for the comparison between NCIT and NCT, stratified by age (<65 or ≥65 years), hypertension (no or yes), pulmonary disease (no or yes), smoking status (no or yes), and clinical tumor stage (I–II or III–IV). Subgroup-specific propensity scores and overlap weights were estimated separately within each stratum.

## 3. Results

A total of 540 patients were included. The median age was 65 years, and the majority were male (385/540, 71.3%). The overall incidence of POP was 36.1% (195/540), with specific rates of 36.9%, 32.3%, 25.0%, and 49.3% in the S-alone, NCT, NCRT, and NCIT groups, respectively. Perioperative characteristics, including operative duration, intraoperative blood loss, postoperative intensive care unit (ICU) admission, and length of ICU stay, are summarized in Table S2. Among the 195 patients who developed POP, 101 (51.8%) received therapeutic antibiotics, with a median treatment duration of 11.0 days; detailed antibiotic treatment data by treatment group are presented in Table S3. Among patients with POP, five (2.6%) required ICU readmission, and no pneumonia-related deaths occurred.

### 3.1. Four-Group Comparison

**Figure 1:**
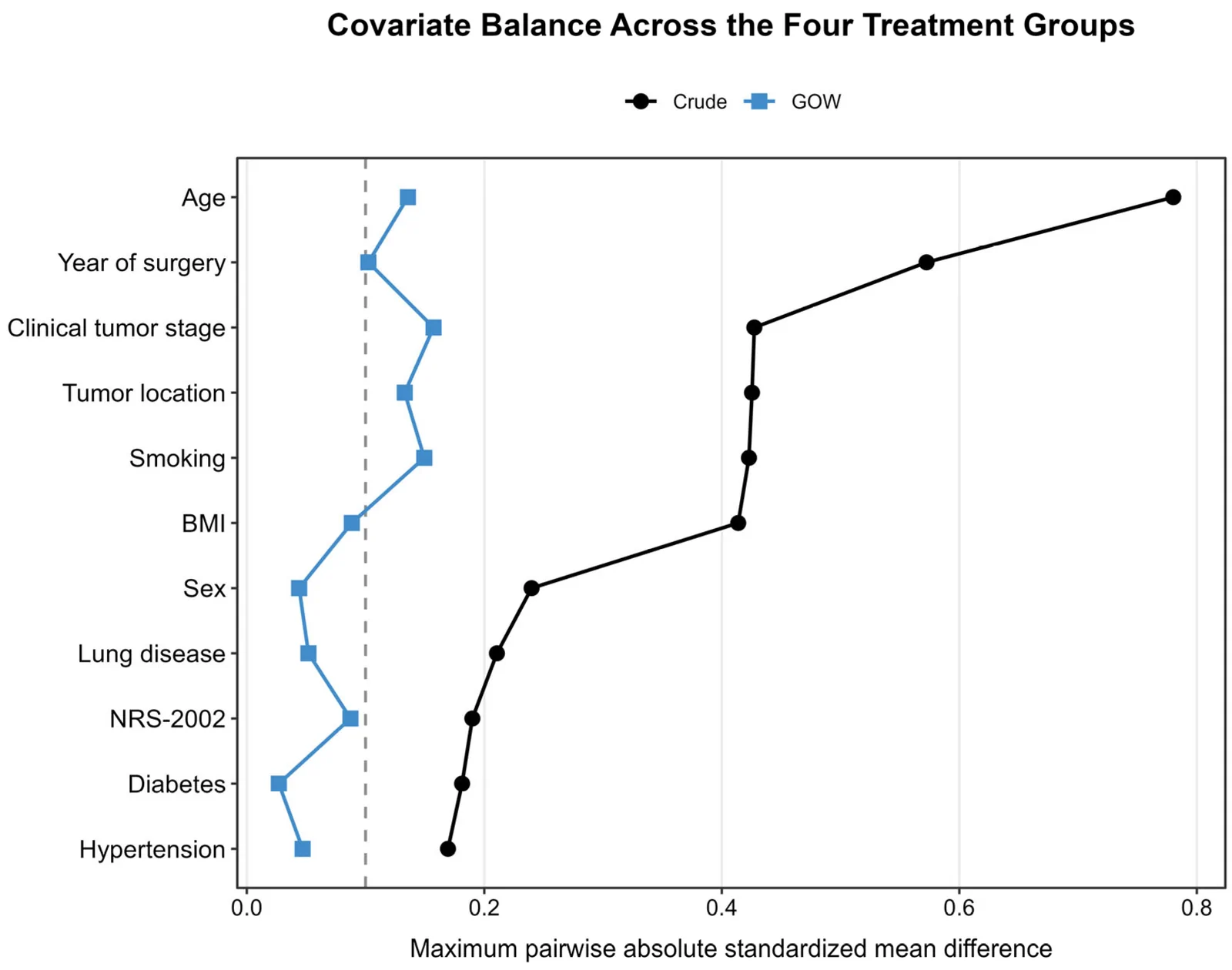
Assessment of covariate balance using GOW in four-group comparison. GOW, generalized overlap weighting; S-alone, surgery alone; NCT, neoadjuvant chemotherapy; NCRT, neoadjuvant chemoradiotherapy; NCIT, neoadjuvant chemoimmunotherapy. The dashed vertical line indicates an SMD of 0.10, the threshold for good covariate balance.

To address potential imbalances, GOW was performed. After weighting, all covariates achieved good or acceptable balance (Figure 1 and Table 1). The results before and after GOW are shown in Table 1. Compared with the S-alone group, the NCIT group had significantly higher odds of POP in Model 3 (*OR*, 1.95; 95% *CI*, 1.02–3.70; *p* = 0.042). No statistically significant differences were observed for the NCT or NCRT groups compared with the S-alone group across the three models (Table 2).

### 3.2. Comparison Between NCRT and NCT

**Figure 2:**
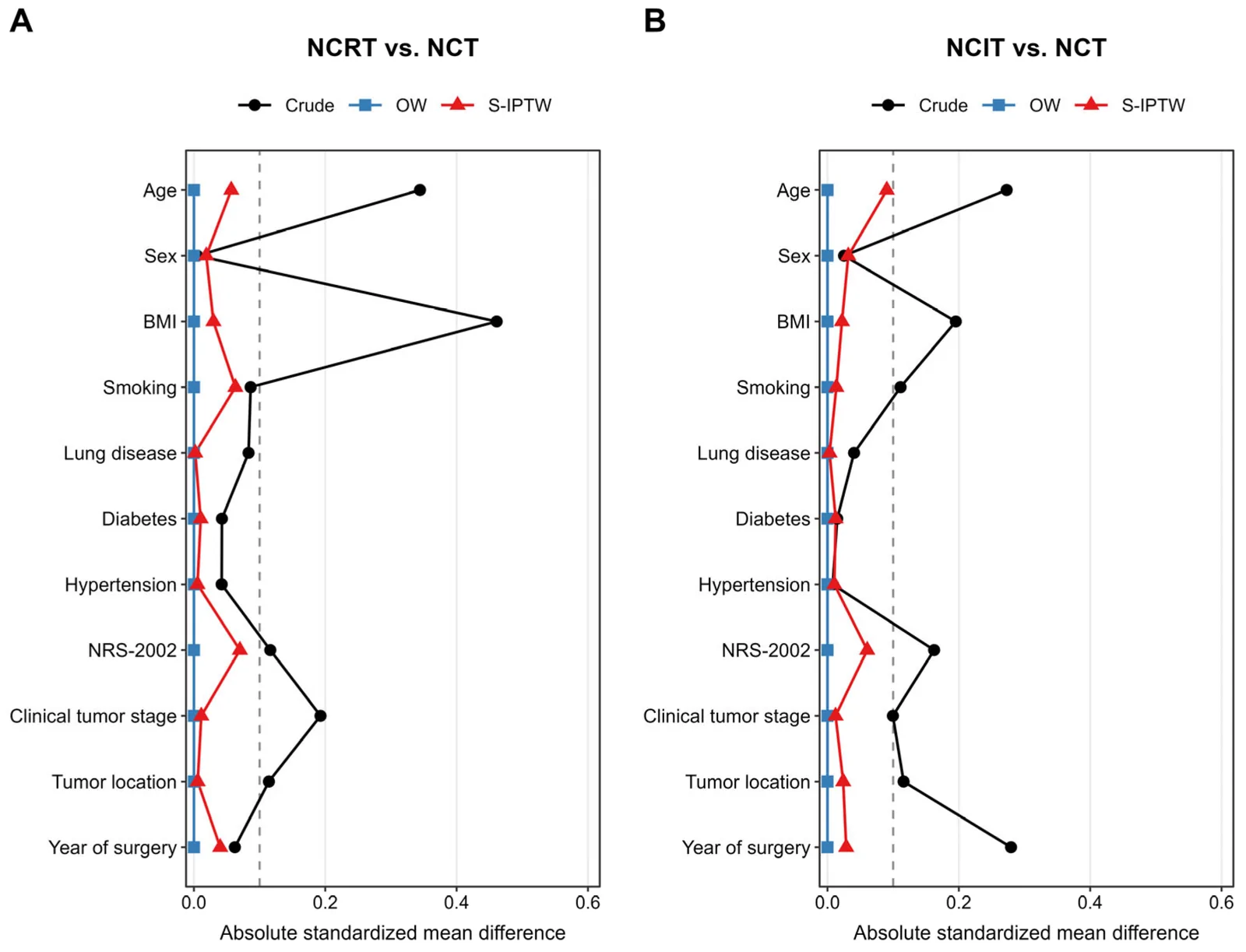
Assessment of covariate balance using different weighting methods in the pairwise comparisons: (**A**) NCRT versus NCT and (**B**) NCIT versus NCT. OW, overlap weighting; S-IPTW, stabilized inverse probability of treatment weighting. The dashed vertical line indicates an SMD of 0.10, the threshold for good covariate balance.

OW performed well at balancing the selected covariates, as evidenced by the improvement in SMD (see Table S4 and Figure 2). No statistically significant difference in POP rate between the NCRT and NCT groups was observed across the three models (all *p* > 0.05; Table 3).

### 3.3. Comparison Between NCIT and NCT

OW achieved excellent covariate balance, with all SMDs below 0.10 between the NCIT and NCT groups (Table S5 and Figure 2). Table 4 shows the NCIT group had higher odds of POP than the NCT group and the OR values in Model 1, 2 and 3 were respectively 2.04 (95% *CI*: 1.13, 3.72), 2.41 (95% *CI*: 1.20, 4.95), and 2.32 (95% *CI*: 1.14, 4.71).

### 3.4. Sensitivity Analysis Results

Tables S6 and S8 present the baseline characteristics and SMDs before and after S-IPTW. Figure 2 shows the SMDs in the original and S-IPTW-weighted data. The results were consistent with those of the primary OW analyses. In the S-IPTW analysis, NCRT was not associated with significantly higher odds of POP than NCT (Table S7), whereas NCIT remained associated with higher odds of POP than NCT (Table S9).

### 3.5. Subgroup Analysis Results

Subgroup analyses showed consistently higher point estimates for NCIT versus NCT across all examined subgroups (Figure S2). Significant associations were observed in patients aged ≥ 65 years, those without hypertension, and those with clinical stage I–II disease. Overall, NCIT was associated with higher odds of POP than NCT.

## 4. Discussion

In this study, GOW and OW were used to address baseline confounding and compare the odds of POP across four treatment strategies. Neither NCT nor NCRT was associated with higher odds of POP than S-alone, and NCRT was not associated with higher odds of POP than NCT. In contrast, NCIT was associated with higher odds of POP than both S-alone and NCT.

Using the S-alone group as a reference for the inherent pulmonary trauma of MIE, our findings aligned with the NewEC study [30], in which neither NCT nor NCRT introduce unacceptable additive pulmonary morbidity. Furthermore, a previous meta-analysis indicated that pulmonary complications were significantly elevated by NCRT compared to NCT [18], conflicting with our finding. However, a Japanese nationwide real-world study concluded postoperative respiratory complications were not significantly increased by NCRT compared to NCT in ESCC patients [31]. This comparable safety was similarly confirmed by Wang et al. in an RCT [14], supporting our result. This difference may partly reflect technological advances. Modern conformal radiotherapy techniques, such as intensity-modulated radiotherapy (IMRT), enable more precise dose delivery, minimize unintended radiation exposure to the lung parenchyma, and may reduce the incidence of radiation pneumonitis [32], while MIE may also improve postoperative pulmonary function and reduce pulmonary complications by minimizing chest wall trauma and preserving respiratory mechanics [10].

Notably, the integration of immunotherapy was associated with higher odds of POP in our cohort. This finding was consistent with a previous propensity score-matched study evaluating NCIT against S-alone [33]. However, recent studies comparing NCIT to traditional regimens have primarily emphasized the efficacy of immunotherapy, reporting no significant increase in perioperative morbidities [34,35], which differs from our findings. These discrepancies might be attributed to inconsistent diagnostic criteria for POP, varying sample sizes, and different confounding adjustment methods (PSM in previous studies versus OW in ours). Nevertheless, several potential mechanisms may partly account for the higher odds of POP observed in the NCIT group. First, immunotherapy could induce severe fibrotic reactions in the tumor and surrounding tissues, which not only prolonged operative time and increased intraoperative blood loss but also complicated lymph node dissection around the airway, exacerbating the surgical stress response [36]. Second, NCIT might provoke immune-related pulmonary inflammation, leaving patients susceptible to overt lung infections when subjected to surgical trauma and general anesthesia [35].

Clinically, the observed association between NCIT and higher odds of POP supports consideration of a risk-adapted perioperative approach rather than changes in oncological treatment decisions based solely on the present findings. Patients receiving NCIT may benefit from careful preoperative assessment of pulmonary comorbidity, respiratory function, nutritional status, smoking history, and overall functional reserve, together with appropriate optimization of reversible respiratory conditions. During the postoperative period, closer surveillance for respiratory deterioration and infection may be considered. Standard preventive measures, including respiratory physiotherapy, airway clearance, early mobilization and aspiration prevention, may also be appropriate. However, whether intensified perioperative respiratory management reduces POP following NCIT requires prospective evaluation.

This study provides real-world evidence on perioperative pulmonary outcomes across different neoadjuvant strategies in ESCC. However, several limitations should be considered. First, its retrospective observational design precludes causal inference, and residual confounding remains possible despite overlap weighting and multivariable adjustment. Although smoking status, nutritional risk assessed using the NRS-2002 score, and calendar period of surgery were included in the analyses, detailed smoking exposure was not fully captured. Second, the proposed mechanisms were not directly evaluated because data on pathological fibrosis, technical difficulty during lymph-node dissection, immune-related adverse events and other pulmonary toxicities were unavailable. Although operative duration and intraoperative blood loss were included in the adjusted analyses, these variables cannot establish a mechanistic pathway. Third, preoperative pulmonary function data were incomplete, and information on standardized respiratory physiotherapy assessment or perioperative respiratory rehabilitation was not systematically recorded. Recurrent laryngeal nerve palsy, postoperative vocal cord dysfunction, and anastomotic leakage were not directly recorded. Postoperative hoarseness was available only as a nonspecific proxy and was too infrequent for reliable multivariable adjustment. Fourth, detailed measures of POP severity, including Clavien–Dindo grade and the need for reintubation or mechanical ventilation, were not consistently documented, although antibiotic treatment was summarized and ICU readmission and pneumonia-related mortality were reported descriptively. Fifth, heterogeneity in chemotherapy regimens, individual PD-1 inhibitors, treatment cycles, treatment-to-surgery intervals, and radiotherapy techniques may have influenced the findings. Regimen-specific analyses were not feasible because of the small and uneven subgroup sizes. Finally, the single-center design limits generalizability, and long-term oncological outcomes were not evaluated. Prospective multicenter studies with standardized perioperative and pulmonary assessments are needed to validate these findings.

## 5. Conclusions

In conclusion, NCRT was not associated with higher odds of POP than surgery alone or NCT, whereas NCIT was associated with higher odds of POP in patients with ESCC undergoing MIE. Given the observational and single-center nature of this study, prospective multicenter studies with standardized perioperative and pulmonary assessments are needed to validate this association. Future studies should also develop and evaluate risk-adapted perioperative respiratory management strategies for patients receiving NCIT.

## Figures and Tables

**Table 1:** Baseline characteristics before and after GOW in the four-group comparison.

	Before Weighting (GOW)						After Weighting (GOW)				
Characteristic	S-Alone (*n* = 282)	NCT (*n* = 127)	NCRT (*n* = 60)	NCIT (*n* = 71)	*p*	SMD	S-Alone (*n* = 198)	NCT (*n* = 106)	NCRT (*n* = 46)	NCIT (*n* = 57)	SMD
Sex					0.097	0.240					0.044
Male	188 (67%)	98 (77%)	46 (77%)	53 (75%)			(74%)	(75%)	(75%)	(73%)	
Female	94 (33%)	29 (23%)	14 (23%)	18 (25%)			(26%)	(25%)	(25%)	(27%)	
Age (year) (median, IQR)	66 (60, 72)	64 (58, 68)	62 (56, 66)	66 (59, 70)	<0.001	0.780	62 (57, 68)	65 (59, 69)	64 (60, 68)	64 (58, 70)	0.136
Year of surgery					0.001	0.572					
2021–2022	135 (48%)	82 (65%)	35 (58%)	26 (37%)			(51%)	(50%)	(46%)	(49%)	0.102
2023–2025	147 (52%)	45 (35%)	25 (42%)	45 (63%)			(49%)	(50%)	(54%)	(51%)	
BMI (kg/m^2^) (median, IQR)	23.60 (21.50, 25.40)	23.70 (22.20, 25.10)	22.40 (20.10, 24.45)	23.20 (21.20, 24.40)	0.024	0.414	22.90 (20.80, 25.00)	23.10 (21.20, 24.70)	22.80 (20.70, 25.90)	22.80 (21.10, 23.90)	0.088
Smoking					0.002	0.423					0.149
No	174 (62%)	66 (52%)	26 (43%)	29 (41%)			(46%)	(46%)	(43%)	(50%)	
Yes	108 (38%)	61 (48%)	34 (57%)	42 (59%)			(54%)	(54%)	(57%)	(50%)	
Lung disease					0.439	0.211					0.052
No	206 (73%)	91 (72%)	38 (63%)	48 (68%)			(65%)	(66%)	(67%)	(65%)	
Yes	76 (27%)	36 (28%)	22 (37%)	23 (32%)			(35%)	(34%)	(33%)	(35%)	
Diabetes					0.702	0.181					0.027
No	252 (89%)	111 (87%)	55 (92%)	61 (86%)			(90%)	(89%)	(90%)	(90%)	
Yes	30 (11%)	16 (13%)	5 (8.3%)	10 (14%)			(10%)	(11%)	(10%)	(10%)	
Hypertension					0.638	0.169					0.047
No	195 (69%)	92 (72%)	46 (77%)	52 (73%)			(74%)	(75%)	(73%)	(74%)	
Yes	87 (31%)	35 (28%)	14 (23%)	19 (27%)			(26%)	(25%)	(27%)	(26%)	
NRS-2002	1.00 (1.00, 2.00)	1.00 (1.00, 2.00)	1.00 (1.00, 2.00)	1.00 (1.00, 2.00)	0.528	0.190	1.00 (1.00, 2.00)	1.00 (1.00, 2.00)	1.00 (1.00, 2.00)	1.00 (1.00, 2.00)	0.087
Clinical tumor stage					0.006	0.427					0.157
I + II	211 (75%)	75 (59%)	47 (78%)	49 (69%)			(74%)	(71%)	(67%)	(73%)	
III + IV	71 (25%)	52 (41%)	13 (22%)	22 (31%)			(26%)	(29%)	(33%)	(27%)	
Tumor location					0.071	0.425					0.133
Upper	34 (12%)	19 (15%)	14 (23%)	6 (8.5%)			(15%)	(14%)	(12%)	(14%)	
Lower	97 (34%)	30 (24%)	16 (27%)	25 (35%)			(33%)	(29%)	(30%)	(29%)	
Middle	151 (54%)	78 (61%)	30 (50%)	40 (56%)			(52%)	(56%)	(58%)	(57%)	

Data are presented as n (%) or median (IQR). Counts in the weighted cohort were rounded. GOW, generalized overlap weighting; S-alone, surgery alone; NCT, neoadjuvant chemotherapy; NCRT, neoadjuvant chemoradiotherapy; NCIT, neoadjuvant chemoimmunotherapy; SMD, standardized mean difference.

**Table 2:** Association between treatment strategy and postoperative pneumonia in the four-group analysis.

	Model 1			Model 2			Model 3		
Groups	*OR*	95% *CI*	*p*	*OR*	95% *CI*	*p*	*OR*	95% *CI*	*p*
S-alone	—	—		—	—		—	—	
NCT	0.82	0.52, 1.27	0.369	0.79	0.48, 1.28	0.340	0.84	0.48, 1.47	0.541
NCRT	0.57	0.29, 1.05	0.082	0.52	0.25, 1.04	0.071	0.49	0.24, 1.02	0.057
NCIT	1.66	0.98, 2.82	0.057	1.64	0.94, 2.87	0.092	1.95	1.02, 3.70	0.042

**Model 1:**  unadjusted model including only the treatment group. **Model 2:** adding sex, age, year of surgery, BMI, smoking, lung disease, diabetes, hypertension, NRS-2002, clinical tumor stage, tumor location, surgical anastomosis, operative duration and intraoperative blood loss on the basis of Model 1. **Model 3:** applying GOW on the basis of Model 2. *OR*, odds ratio; *CI*, confidence interval; S-alone, surgery alone; NCT, neoadjuvant chemotherapy; NCRT, neoadjuvant chemoradiotherapy; NCIT, neoadjuvant chemoimmunotherapy.

**Table 3:** Association between treatment group and postoperative pneumonia in the NCRT versus NCT comparison using OW.

	Model 1			Model 2			Model 3		
Groups	*OR*	95% *CI*	*p*	*OR*	95% *CI*	*p*	*OR*	95% *CI*	*p*
NCT	—	—		—	—		—	—	
NCRT	0.70	0.34, 1.38	0.311	0.45	0.18, 1.01	0.062	0.45	0.20, 1.01	0.054

**Model 1:** unadjusted model containing only the treatment group. **Model 2:** adding sex, age, year of surgery, BMI, smoking, lung disease, diabetes, hypertension, NRS-2002, clinical tumor stage, tumor location, surgical anastomosis, operative duration and intraoperative blood loss on the basis of Model 1. **Model 3:** applying OW on the basis of Model 2. *OR*, odds ratio; *CI*, confidence interval; NCT, neoadjuvant chemotherapy; NCRT, neoadjuvant chemoradiotherapy.

**Table 4:** Association between treatment group and postoperative pneumonia in the NCIT versus NCT comparison using OW.

	Model 1			Model 2			Model 3		
Group	*OR*	95% *CI*	*p*	*OR*	95% *CI*	*p*	*OR*	95% *CI*	*p*
NCT	—	—		—	—		—	—	
NCIT	2.04	1.13, 3.72	0.019	2.41	1.20, 4.95	0.015	2.32	1.14, 4.71	0.020

**Model 1:** unadjusted model containing only the treatment group. **Model 2:** adding sex, age, year of surgery, BMI, smoking, lung disease, diabetes, hypertension, NRS-2002, clinical tumor stage, tumor location, surgical anastomosis, operative duration and intraoperative blood loss on the basis of Model 1. **Model 3:** applying OW on the basis of Model 2. *OR*, odds ratio; *CI*, confidence interval; NCT, neoadjuvant chemotherapy; NCIT, neoadjuvant chemoimmunotherapy.

## Data Availability

The raw data supporting the conclusions of this article will be made available by the authors on request.

## Supplementary Materials

The following supporting information can be downloaded at: https://www.mdpi.com/article/10.3390/cancers18162571/s1. Table S1: Detailed characteristics of neoadjuvant treatments; Table S2: Perioperative characteristics by treatment group; Table S3: Antibiotic treatment among patients with postoperative pneumonia; Table S4: Baseline characteristics before and after OW in NCT and NCRT; Table S5: Baseline characteristics before and after OW in NCT and NCIT; Table S6: Baseline characteristics before and after S-IPTW in NCT and NCRT; Table S7: Association between treatment strategy and postoperative pneumonia in the NCRT versus NCT comparison using S-IPTW; Table S8: Baseline characteristics before and after S-IPTW in NCT and NCIT; Table S9: Association between treatment strategy and postoperative pneumonia in the NCIT versus NCT comparison using S-IPTW; Figure S1. Flow diagram of the study. ESCC esophageal squamous cell carcinoma, MIE minimally invasive esophagectomy, S-alone surgery alone, NCT neoadjuvant chemotherapy, NCRT neoadjuvant chemoradiotherapy, NCIT neoadjuvant chemoimmunotherapy. Figure S2. Subgroup analysis of postoperative pneumonia comparing NCIT with NCT. OR, odds ratio; CI, confidence interval; NCIT, neoadjuvant chemoimmunotherapy; NCT, neoadjuvant chemotherapy.

## Abbreviations

The following abbreviations are used in this manuscript:

POP	postoperative pneumonia
MIE	minimally invasive esophagectomy
ESCC	esophageal squamous cell carcinoma
S-alone	surgery alone
NCT	neoadjuvant chemotherapy
NCRT	neoadjuvant chemoradiotherapy
NCIT	neoadjuvant chemoimmunotherapy
GOW	generalized overlap weighting
OW	overlap weighting
S-IPTW	stabilized inverse probability of treatment weighting
OR	odds ratio
CI	confidence interval
EC	esophageal cancer
OS	overall survival
BMI	body mass index
NRS-2002	Nutritional Risk Screening 2002
PD-1	programmed cell death protein 1
ERAS	enhanced recovery after surgery
ICU	intensive care unit
RCT	randomized controlled trial
PSM	propensity score matching
IPTW	inverse probability of treatment weighting
IQR	interquartile range
SMD	standardized mean difference
IMRT	intensity-modulated radiotherapy
CT	computed tomography
