# Supplementary material for: Postoperative Pneumonia After Minimally Invasive Esophagectomy in Esophageal Squamous Cell Carcinoma: A Weighted Real-World Comparison of Neoadjuvant Treatment Strategies
Source: Cancers (Basel). 2026 Aug 10;18(16):2571. doi: 10.3390/cancers18162571 (PMC13511234; doi:10.3390/cancers18162571)
Supplement: Supplementary file 1 [file cancers-18-02571-s001.zip › cancers-4439123-supplementary.pdf]

SUPPLEMENTARY MATERIALS

Tables

Table S1. Detailed characteristics of neoadjuvant treatments

Characteristics	Total Treated Cohort
**Chemotherapy Regimens (N = 258)**	
Paclitaxel + Platinum (Cisplatin, Carboplatin, or Nedaplatin)	213 (82.6%)
Docetaxel + Nedaplatin	13 (5.0%)
Others	32 (12.4%)
**PD-1 Inhibitors for NCIT group (N = 71)**	
Toripalimab	24 (33.8%)
Sintilimab	22 (31.0%)
Camrelizumab	18 (25.4%)
Tislelizumab	6 (8.5%)
Others	1 (1.4%)
**Radiotherapy Parameters for NCRT group (N = 60)**	
Prescribed dose: 41.4 Gy / 23 fractions	52 (86.7%)
Other doses	8 (13.3%)
**Median systemic treatment cycles (IQR)**	
NCT group (Chemotherapy cycles)	3 (1, 5)
NCIT group (Concurrent chemo-immunotherapy cycles)	3.5 (2, 5)
NCRT group (Concurrent chemotherapy cycles)	3 (1, 4)

Table S2. Perioperative characteristics by treatment group

Groups	Overall N=540	S-alone N=282	NCT N=127	NCRT N=60	NCIT N=71	*P*
Operative duration, min	276.0 (236.0, 326.0)	263.5 (221.0, 310.0)	289.0 (240.0, 330.0)	298.5 (240.5, 340.0)	294.0 (246.0, 331.0)	0.006
Intraoperative blood loss, mL	100.0 (100.0, 150.0)	100.0 (80.0, 150.0)	100.0 (100.0, 200.0)	100.0 (90.0, 200.0)	100.0 (100.0, 150.0)	0.047
Postoperative ICU admission						0.500
No	294 (54%)	159 (56%)	71 (56%)	30 (50%)	34 (48%)	
Yes	246 (46%)	123 (44%)	56 (44%)	30 (50%)	37 (52%)	
Length of ICU stay among ICU-admitted patients, days	2.0 (2.0, 2.0)	2.0 (2.0, 2.0)	2.0 (2.0, 2.0)	2.0 (2.0, 2.0)	2.0 (2.0, 3.0)	0.11

Values are presented as median (interquartile range) or n (%). P values were calculated using the Kruskal-Wallis test for continuous variables and the chi-square test for categorical variables. ICU, intensive care unit.

Table S3. Antibiotic treatment among patients with postoperative pneumonia

Groups	Overall	S-alone	NCT	NCRT	NCIT	*P*
Postoperative pneumonia cases, n	195	104	41	15	35	
Antibiotic therapy, n (%)	101 (51.8%)	57 (54.8%)	14 (34.1%)	7 (46.7%)	23 (65.7%)	0.039
Antibiotic duration, days, median (IQR)	11.0 (7.0,16.0)	12.0 (7.0,17.0)	10.5 (5.0,19.0)	7.0 (1.0,11.0)	9.0 (6.0,13.0)	0.090

Values are presented as n (%) or median (interquartile range). Percentages for antibiotic therapy use the number of patients with postoperative pneumonia in each treatment group as the denominator. Antibiotic duration was summarized only among patients who received antibiotic therapy. P values were calculated using the chi-square test and the Kruskal-Wallis test, as appropriate.

Table S4. Baseline characteristics before and after OW in NCT and NCRT

Characteristic	**Before weighting (OW)** NCT (n = 127)	NCRT (n = 60)	*P*	SMD	**After weighting (OW)** NCT (n = 101)	NCRT (n = 53)	SMD
Sex			>0.999	0.012			0.000
Male	98 (77%)	46 (77%)			(77%)	(77%)	
Female	29 (23%)	14 (23%)			(23%)	(23%)	
Age (year) (median, IQR)	64 (58, 68)	62 (56, 66)	0.038	0.334	63 (58, 68)	64 (58, 67)	0.000
Year of surgery			0.509	0.128			0.000
2021-2022	82 (65%)	35 (58%)			(58%)	(58%)	
2023-2025	45 (35%)	25 (42%)			(42%)	(42%)	
BMI (kg/m2) (median, IQR)	23.70 (22.20, 25.10)	22.40 (20.10, 24.45)	0.005	0.444	23.10 (21.20, 24.60)	22.80 (21.10, 25.00)	0.000
Smoking			0.344	0.174			0.000
No	66 (52%)	26 (43%)			(45%)	(45%)	
Yes	61 (48%)	34 (57%)			(55%)	(55%)	
Lung disease			0.328	0.178			0.000

No	91 (72%)	38 (63%)			(67%)	(67%)	
Yes	36 (28%)	22 (37%)			(33%)	(33%)	
Diabetes			0.539	0.140			0.000
No	111 (87%)	55 (92%)			(91%)	(91%)	
Yes	16 (13%)	5 (8.3%)			(9%)	(9%)	
Hypertension			0.663	0.097			0.000
No	92 (72%)	46 (77%)			(76%)	(76%)	
Yes	35 (28%)	14 (23%)			(24%)	(24%)	
NRS-2002	1.00 (1.00, 2.00)	1.00 (1.00, 2.00)	0.912	0.111	1.00 (1.00, 2.00)	1.00 (1.00, 2.00)	0.000
Clinical tumor stage			0.016	0.425			0.000
I + II	75 (59%)	47 (78%)			(71%)	(71%)	
III + IV	52 (41%)	13 (22%)			(29%)	(29%)	
Tumor location			0.260	0.231			0.000
Upper	19 (15%)	14 (23%)			(20%)	(20%)	
Lower	30 (24%)	16 (27%)			(24%)	(24%)	
Middle	78 (61%)	30 (50%)			(56%)	(56%)	

Data are presented as n (%) or median (IQR). Counts in the weighted cohort were rounded.

OW, overlap weighting; NCT, neoadjuvant chemotherapy; NCRT, neoadjuvant chemoradiotherapy; SMD, standardized mean differences.

Table S5. Baseline characteristics before and after OW in NCT and NCIT

Characteristic	Before weighting (OW)				After weighting (OW)		
	NCT (n = 127)	NCIT (n = 71)	*P*	SMD	NCT (n = 106)	NCIT (n = 66)	SMD
Sex			0.822	0.059			0.000
Male	98 (77%)	53 (75%)			(75%)	(75%)	
Female	29 (23%)	18 (25%)			(25%)	(25%)	
Age (year) (median, IQR)	64 (58, 68)	66 (59, 70)	0.061	0.263	66 (59, 69)	66 (59, 70)	0.000
Year of surgery			< 0.001	0.582			0.000
2021-2022	82 (65%)	26 (37%)			(47%)	(47%)	
2023-2025	45 (35%)	45 (63%)			(53%)	(53%)	
BMI (kg/m2) (median, IQR)	23.70 (22.20, 25.10)	23.20 (21.20, 24.40)	0.094	0.198	23.40 (21.70, 24.80)	23.30 (21.30, 24.70)	0.000
Smoking			0.176	0.224			0.000
No	66 (52%)	29 (41%)			(47%)	(47%)	
Yes	61 (48%)	42 (59%)			(53%)	(53%)	
Lung disease			0.663	0.088			0.000
No	91 (72%)	48 (68%)			(68%)	(68%)	
Yes	36 (28%)	23 (32%)			(32%)	(32%)	
Diabetes			0.938	0.044			0.000
No	111 (87%)	61 (86%)			(86%)	(86%)	
Yes	16 (13%)	10 (14%)			(14%)	(14%)	
Hypertension			>0.999	0.018			0.000
No	92 (72%)	52 (73%)			(73%)	(73%)	
Yes	35 (28%)	19 (27%)			(27%)	(27%)	

NRS-2002	1.00 (1.00, 2.00)	1.00 (1.00, 2.00)	0.280	0.166	1.00 (1.00, 2.00)	1.00 (1.00, 2.00)	0.000
Clinical tumor stage			0.216	0.209			0.000
I + II	75 (59%)	49 (69%)			(66%)	(66%)	
III + IV	52 (41%)	22 (31%)			(34%)	(34%)	
Tumor location			0.140	0.256			0.000
Upper	19 (15%)	6 (8.5%)			(10%)	(10%)	
Lower	30 (24%)	25 (35%)			(31%)	(31%)	
Middle	78 (61%)	40 (56%)			(58%)	(58%)	

Data are presented as n (%) or median (IQR). Counts in the weighted cohort were rounded.

OW, overlap weighting; NCT, neoadjuvant chemotherapy; NCIT, neoadjuvant chemoimmunotherapy; SMD, standardized mean differences.

Table S6. Baseline characteristics before and after S-IPTW in NCT and NCRT

	Before weighting (S-IPTW)				After weighting (S-IPTW)		
**Characteristic**	**NCT (n = 127)**	**NCRT (n = 60)**	***P***	**SMD**	**NCT (n = 120)**	**NCRT (n = 42)**	**SMD**
Sex			>0.999	0.012			0.045
Male	98 (77%)	46 (77%)			(78%)	(79%)	
Female	29 (23%)	14 (23%)			(22%)	(21%)	
Age (year) (median, IQR)	64 (58, 68)	62 (56, 66)	0.038	0.334	63 (58, 68)	64 (59, 68)	0.057
Year of surgery			0.509	0.128			0.082
2021-2022	82 (65%)	35 (58%)			(62%)	(58%)	
2023-2025	45 (35%)	25 (42%)			(38%)	(42%)	
BMI (kg/m2) (median, IQR)	23.70 (22.20, 25.10)	22.40 (20.10, 24.45)	0.005	0.444	23.50 (21.70, 24.80)	23.00 (21.90, 26.00)	0.030
Smoking			0.344	0.174			0.127
No	66 (52%)	26 (43%)			(49%)	(42%)	
Yes	61 (48%)	34 (57%)			(51%)	(58%)	
Lung disease			0.328	0.178			0.004
No	91 (72%)	38 (63%)			(70%)	(70%)	
Yes	36 (28%)	22 (37%)			(30%)	(30%)	
Diabetes			0.539	0.140			0.034
No	111 (87%)	55 (92%)			(89%)	(90%)	
Yes	16 (13%)	5 (8.3%)			(11%)	(10%)	
Hypertension			0.663	0.097			0.013
No	92 (72%)	46 (77%)			(74%)	(74%)	
Yes	35 (28%)	14 (23%)			(26%)	(26%)	
NRS-2002	1.00 (1.00, 2.00)	1.00 (1.00, 2.00)	0.912	0.111	1.00 (1.00, 2.00)	1.00 (1.00, 2.00)	0.070
Clinical tumor stage			0.016	0.425			0.025
I + II	75 (59%)	47 (78%)			(64%)	(65%)	
III + IV	52 (41%)	13 (22%)			(36%)	(35%)	
Tumor location			0.260	0.231			0.013
Upper	19 (15%)	14 (23%)			(18%)	(17%)	
Lower	30 (24%)	16 (27%)			(24%)	(25%)	

Middle	78 (61%)	30 (50%)			(58%)	(58%)

Data are presented as n (%) or median (IQR). Counts in the weighted cohort were rounded.

S-IPTW, stabilized inverse probability of treatment weighting; NCT, neoadjuvant chemotherapy; NCRT, neoadjuvant chemoradiotherapy; SMD, standardized mean differences.

Table S7. Association between treatment strategy and postoperative pneumonia in the NCRT versus NCT comparison using S-IPTW

	Model 1			Model 2			Model 3		
**Group**	**OR**	**95% CI**	***P***	**OR**	**95% CI**	***P***	**OR**	**95% CI**	***P***
NCT	—	—		—	—		—	—	
NCRT	0.70	0.34, 1.38	0.311	0.45	0.18, 1.01	0.063	0.48	0.20, 1.18	0.108

Model 1: unadjusted model containing only the treatment group.

Model 2: adding sex, age, year of surgery, BMI, smoking, lung disease, diabetes, hypertension, NRS-2002, clinical tumor stage, tumor location, surgical anastomosis, operative duration and intraoperative blood loss on the basis of Model 1.

Model 3: applying S-IPTW on the basis of Model 2.

OR, odds ratio; CI, confidence interval; NCT, neoadjuvant chemotherapy; NCRT, neoadjuvant chemoradiotherapy.

Table S8. Baseline characteristics before and after S-IPTW in NCT and NCIT

	Before weighting (S-IPTW)				After weighting (S-IPTW)		
**Characteristic**	**NCT (n = 127)**	**NCIT (n = 71)**	***P***	**SMD**	**NCT (n = 120)**	**NCIT (n = 53)**	**SMD**
Sex			0.822	0.059			0.074
Male	98 (77%)	53 (75%)			(76%)	(73%)	
Female	29 (23%)	18 (25%)			(24%)	(27%)	
Age(year) (median, IQR)	64 (58, 68)	66 (59, 70)	0.061	0.263	65 (59, 69)	65 (59, 70)	0.090
Year of surgery			＜ 0.001	0.582			0.059
2021-2022	82 (65%)	26 (37%)			(54%)	(51%)	
2023-2025	45 (35%)	45 (63%)			(46%)	(49%)	
BMI (kg/m2) (median, IQR)	23.70 (22.20, 25.10)	23.20 (21.20, 24.40)	0.094	0.198	23.50 (21.80, 25.00)	23.30 (21.30, 25.10)	0.022
Smoking			0.176	0.224			0.027
No	66 (52%)	29 (41%)			(49%)	(50%)	
Yes	61 (48%)	42 (59%)			(51%)	(50%)	
Lung disease			0.663	0.088			0.007
No	91 (72%)	48 (68%)			(70%)	(70%)	
Yes	36 (28%)	23 (32%)			(30%)	(30%)	
Diabetes			0.938	0.044			0.037
No	111 (87%)	61 (86%)			(86%)	(87%)	
Yes	16 (13%)	10 (14%)			(14%)	(13%)	
Hypertension			>0.999	0.018			0.023
No	92 (72%)	52 (73%)			(72%)	(71%)	
Yes	35 (28%)	19 (27%)			(28%)	(29%)	

NRS-2002	1.00 (1.00, 2.00)	1.00 (1.00, 2.00)	0.280	0.166	1.00 (1.00, 2.00)	1.00 (1.00, 2.00)	0.061
Clinical tumor stage			0.216	0.209			0.026
I + II	75 (59%)	49 (69%)			(64%)	(65%)	
III + IV	52 (41%)	22 (31%)			(36%)	(35%)	
Tumor location			0.140	0.256			0.075
Upper	19 (15%)	6 (8.5%)			(13%)	(11%)	
Lower	30 (24%)	25 (35%)			(29%)	(30%)	
Middle	78 (61%)	40 (56%)			(58%)	(59%)	

Data are presented as n (%) or median (IQR). Counts in the weighted cohort were rounded.

S-IPTW, stabilized inverse probability of treatment weighting; NCT, neoadjuvant chemotherapy; NCIT, neoadjuvant chemoimmunotherapy.

Table S9. Association between treatment strategy and postoperative pneumonia in the NCIT versus NCT comparison using S-IPTW

	**Model 1**			**Model 2**			**Model 3**		
**Group**	**OR**	**95% CI**	***P***	**OR**	**95% CI**	***P***	**OR**	**95% CI**	***P***
NCT	—	—		—	—		—	—	
NCIT	2.04	1.13, 3.72	0.019	2.38	1.17, 4.93	0.018	2.09	1.03, 4.27	0.042

Model 1: unadjusted model containing only the treatment group.

Model 2: adding sex, age, year of surgery, BMI, smoking, lung disease, diabetes, hypertension, NRS-2002, clinical tumor stage, tumor location, surgical anastomosis, operative duration and intraoperative blood loss on the basis of Model 1.

Model 3: applying S-IPTW on the basis of Model 2.

OR, odds ratio; CI, confidence interval; NCT, neoadjuvant chemotherapy; NCIT, neoadjuvant chemoimmunotherapy.

Figures

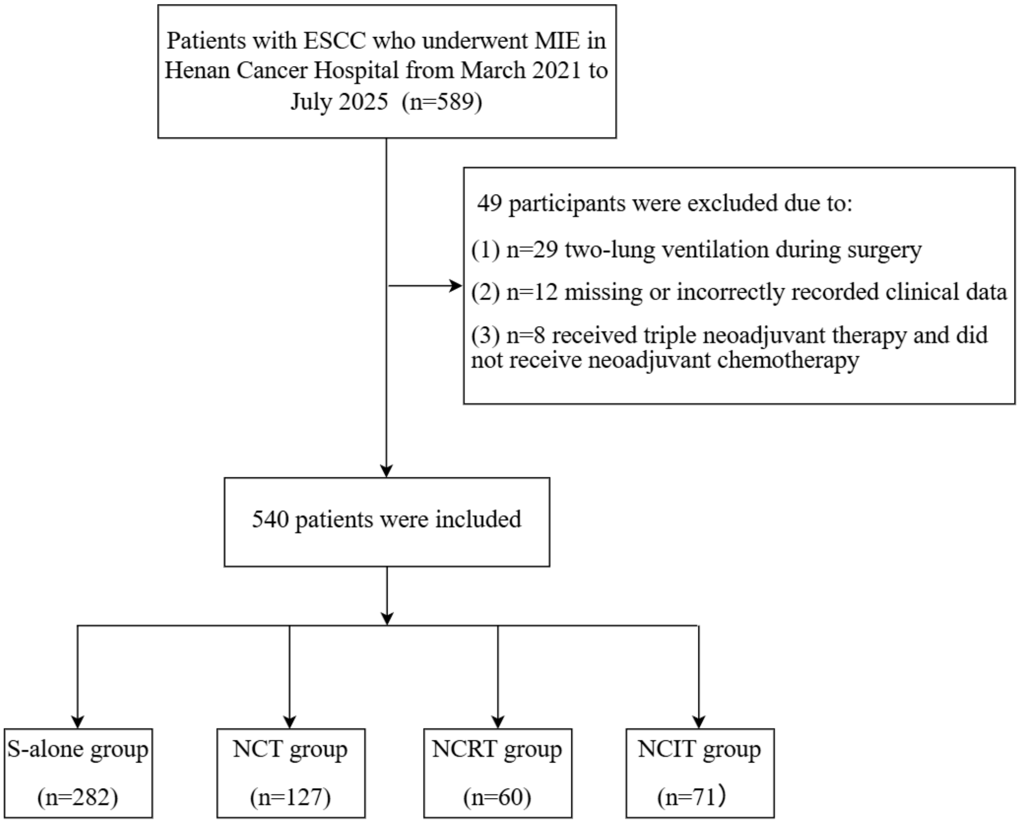

Figure S1. Flow diagram of the study. ESCC esophageal squamous cell carcinoma, MIE minimally invasive esophagectomy, S-alone surgery alone, NCT neoadjuvant chemotherapy, NCRT neoadjuvant chemoradiotherapy, NCIT neoadjuvant chemoimmunotherapy.

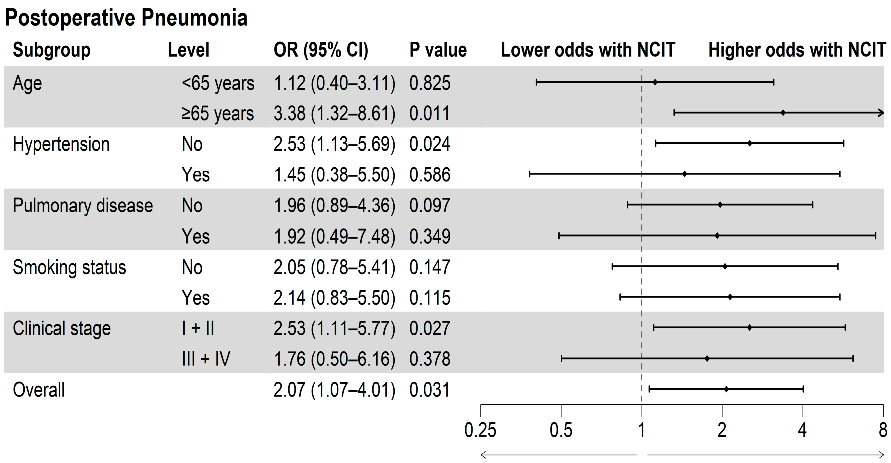

Figure S2. Subgroup analysis of postoperative pneumonia comparing NCIT with NCT. OR, odds ratio; CI, confidence interval; NCIT, neoadjuvant chemoimmunotherapy; NCT,

neoadjuvant chemotherapy.
